# Bioactive Peptide C248 of PRDX4 Ameliorates the Function of Testicular Leydig Cells via Mitochondrial Protection

**DOI:** 10.3390/antiox15010021

**Published:** 2025-12-22

**Authors:** Nini Wei, Shuning Yuan, Li Gao, Bei Zhang, Zhengjie Yan, Chao Gao, Yan Meng, Yugui Cui

**Affiliations:** State Key Laboratory of Reproductive Medicine and Offspring Health, Clinical Center of Reproductive Medicine, The First Affiliated Hospital with Nanjing Medical University, Nanjing 210029, China; weinini@stu.njmu.edu.cn (N.W.); snyuan97@stu.njmu.edu.cn (S.Y.); gaoli_gao@stu.njmu.edu.cn (L.G.); zhangbei1990@suda.edu.cn (B.Z.); yanzhengjie@jsph.org.cn (Z.Y.); gaochao@jsph.org.cn (C.G.); ctmengyan@njmu.edu.cn (Y.M.)

**Keywords:** LCs senescence, antioxidant peptide, peroxiredoxin 4, nicotinamide mononucleotide, mitochondrial protection

## Abstract

Background: The senescence of testicular Leydig cells (LCs) is a key cause of age-related testosterone deficiency, in which oxidative stress (OS) and mitochondrial dysfunction are critical driving mechanisms. We explore whether the bioactive peptide C248 of PRDX4, an intracellular antioxidant, exerts mitochondrial protection to ameliorate LCs’ function. Methods: Based on the antioxidant domains of the PRDX4 protein, small molecular peptides were designed, and bioactive peptide C248 stood out from the crowd. An OS-induced senescence model of LCs was constructed by treating the MLTC-1 cell line with hydrogen peroxide (H_2_O_2_). C248 peptide or nicotinamide mononucleotide (NMN), as the positive control, was administered in the culture medium. The cellular function-related indicators, including DPPH free radical scavenging rate, cell viability, testosterone level, hydrogen peroxide (H_2_O_2_) content, senescence-associated β-galactosidase (SA-β-gal) activity, 8-hydroxy-2′-deoxyguanosine (8-OHDG) level, and 4-hydroxynonenal (4-HNE) level, were evaluated. The mitochondrial function and structural indicators, such as mitochondrial membrane potential, ATP production, mitochondrial morphology, and mitochondrial DNA (mtDNA) copy number, were subsequently tested. Results: In vitro experiments confirmed that C248 could scavenge DPPH free radicals in a dose-dependent manner, reduce the levels of reactive oxygen species, and increase antioxidant enzyme activity in LCs (*p* < 0.01). Both C248 and NMN increased testosterone secretion and improved cell viability (*p* < 0.01). Both C248 and NMN increased mitochondrial morphology and quantity, mitochondrial membrane potential (*p* < 0.01), ATP production (*p* < 0.01), and mitochondrial DNA (mtDNA) copy number (*p* < 0.01). Conclusion: This study reveals that the small molecular C248, a bioactive peptide of PRDX4, is a new candidate molecule for intervening in LC senescence and confirms that mitochondrial protection is a key strategy for improving age-related testicular dysfunction.

## 1. Introduction

Androgens secreted by testicular LCs play a pivotal role in regulating male growth and development, spermatogenesis, and reproductive function throughout the entire life cycle [1]. With advancing age, the testes undergo degenerative changes, among which LCs exhibit a characteristic of senescence susceptibility [2,3]. Cellular senescence is traditionally described as a disability or disorder when cells are exposed to stress or damage, leading to various changes in cell traits [4]. Aging of LCs is characterized by three key features: reduced cell number, decreased responsiveness to luteinizing hormone (LH), and diminished androgen synthesis capacity. These age-associated changes are closely associated with reproductive system disorders in middle-aged and elderly men, named as late-onset hypogonadism (LOH) [3,5,6]. In the mouse model of testicular oxidative stress, a significant decrease in serum testosterone level has been consistently observed, accompanied by a concurrent decline in sperm motility [7]. Accumulating evidence indicates that the testes are more vulnerable to oxidative damage compared to other tissues, and LCs, in particular, show heightened sensitivity to oxidative stress [8,9]. The senescence of LCs primarily stems from the oxidative stress responses induced by reactive oxygen species (ROS). Notably, LCs generate substantial amounts of ROS during the process of testosterone biosynthesis, which further enhances their vulnerability to ROS-mediated damage. This may be one of the main causes of LOH [3,10,11,12]. Given the critical impact of LC senescence on male reproductive health, it is urgent to explore reliable strategies to mitigate oxidative stress-induced senescence and its associated pathological consequences.

The core pathological feature of LC senescence is a vicious cycle between oxidative stress and mitochondrial dysfunction [13]. Mitochondria serve as the primary site for testosterone biosynthesis, providing both key enzymes and substantial adenosine triphosphate (ATP), essential for this biosynthetic process. Concurrently, the mitochondrial respiratory chain plays a pivotal role in regulating intracellular redox homeostasis [14]. Under oxidative stress conditions, excessive ROS directly damage mitochondria, and then oxidative modifications to mitochondrial DNA (mtDNA) impair the expression of respiratory chain complexes, thereby reducing oxidative phosphorylation efficiency [15]. In addition, the peroxidation of mitochondrial membrane phospholipids leads to the collapse of mitochondrial membrane potential, causing abnormal mitophagy and disordered cristae structure [16,17,18]. Such mitochondrial damage further increases ROS leakage, forming a self-reinforcing cycle that ultimately compromises the function of LCs. Notably, mitochondrial redox balance directly modulates the activity of steroidogenic enzymes. Excessive ROS oxidizes critical cysteine residues in the cytochrome P450 side-chain cleavage enzyme (CYP11A1) and 3β-hydroxysteroid dehydrogenase (3β-HSD), resulting in their inactivation [12]. Collectively, these findings underscore that mitochondria are not only the functional core of LCs but also a sensitive target of oxidative stress, highlighting their central role in mediating age-related dysfunction of LCs.

To break the detrimental cycle between oxidative stress and mitochondrial dysfunction, we focused on two complementary intervention strategies: reducing mitochondrial damage at the source by scavenging ROS and repairing impaired mitochondrial function by enhancing energy metabolism. (1) Nicotinamide mononucleotide (NMN) is the direct precursor of nicotinamide adenine dinucleotide (NAD^+^) [19]. Supplementation with NMN activates the deacetylase Sirtuin 3 (Sirt3). This activation enhances the activity of superoxide dismutase 2 (SOD2) and improves oxidative phosphorylation efficiency [20]. Notably, NMN exerts its protective effects by restoring mitochondrial energy metabolism rather than directly scavenging ROS. (2) Antioxidant peptides have gained increasing attention in food research, pharmaceutical research, and other research fields [21]. The antioxidant peptide sequence SFRWQ, as an example identified from medicinal plants, can protect cells from oxidative stress after chemical synthesis [22]. Additionally, a mitochondria-targeted small peptide named SS-31 can stabilize mitochondrial lipids and thereby prevent the aberrant production of ROS [23]. Notably, our previous studies demonstrated that Peroxiredoxin 4 (PRDX4) is a key antioxidant protein in the reproductive system, enabling self-repair of the ovary and testis under conditions of stress and injury [24,25]. Mechanistically, PRDX4 maintains redox homeostasis by scavenging hydrogen peroxide (H_2_O_2_), thereby playing a critical role in alleviating oxidative stress [26].

Based on the protein structure of PRDX4, we designed a group of small peptides that mimics the conserved antioxidant domains (such as a peptide C248 with amino acid sequence YTDKHGEVCPAG), with the aim of investigating the intracellular roles in combating oxidative stress of LCs. As described above, NMN can be used as an ideal positive control for the roles of PRDX4-derived peptide C248. By detecting its antioxidant activity and its effects on the viability and testosterone secretion of LCs senesced by oxidative stress, we revealed that C248 has good antioxidant activity. We also found that both C248 and NMN ultimately alleviate LC senescence by restoring mitochondrial morphology, number, and function. Collectively, this study provides a novel peptide as a candidate for intervening in LC senescence and confirms that mitochondrial protection is a common mechanism through which different anti-aging molecules exert beneficial effects.

## 2. Materials and Methods

### 2.1. Peptide Design

Human PRDX4 and murine Prdx4 protein sequences were retrieved from the National Center for Biotechnology Information (https://www.ncbi.nlm.nih.gov/, accessed on 8 April 2022). Peptides were synthesized via Fmoc chemistry using a Shimadzu PSSM-8 automated solid-phase peptide synthesizer and analyzed by Agilent 110 automated HPLC (Table 1).

### 2.2. Peptide C248 Tracing

A Fam fluorescent tag was conjugated to the target peptide for tracing. Logarithmic phase cells were seeded in confocal dishes; after adhesion, the medium was replaced with one containing fluorescently labeled peptides and incubated at 37 °C with 5% CO_2_ for 4 h. The medium was discarded, and the cells were fixed with 4% paraformaldehyde for 10 min and washed 3× with PBS (5 min each). The cells were stained with Hoechst 33342 (Invitrogen, Carlsbad, CA, USA, Cat. No. 2747528) in the dark for 30 min, then washed 3× with PBS (5 min each). Antifluorescence quenching mounting medium was added for sealing, and the samples were observed/imaged under a confocal microscope (Leica STELLARIS, Wetzlar, Germany; Fam: 488 nm excitation/520 nm emission; Hoechst 33342: 350 nm excitation/460 nm emission).

### 2.3. Cell Culture and Treatment

Mouse Leydig tumor cells (MLTC-1) were purchased from the American Type Culture Collection (ATCC). The MLTC-1 cells were cultured in DMEM/1640 medium (Gibco, New York, NY, USA, Cat. No. 11885084) supplemented with 10% fetal bovine serum (Gibco, Cat. No. A5256701) and 1% penicillin–streptomycin solution (HCM, Cat. No. C100C5). The cells were maintained in a humidified incubator at 37 °C with 5% CO_2_. When the cell confluence reached 80%, they were subcultured using 0.25% trypsin–EDTA solution (HCM, Cat. No. C100C1).

To establish a model of oxidative stress-induced cellular senescence, the cells were treated with 100 μM hydrogen peroxide (H_2_O_2_, Sigma-Aldrich, St. Louis, MO, USA, Cat. No. H1009) for 24 h. Both C248 and NMN were originally stored as dry powders. Double-distilled water (ddH_2_O) was used as the solvent to prepare stock solutions, with concentrations of 1 mM for C248 and 10 mM for NMN. In the intervention experiments, the cells were treated as follows: (1) Pep-C248 group. The cells were cotreated with C248 at a concentration of 50 nM and H_2_O_2_ for 24 h. (2) NMN group. The cells were pretreated with 500 μM NMN (MedChemExpress, Monmouth Junction, NJ, USA, Cat. No. HY—12941) for 2 h and then treated with H_2_O_2_.

### 2.4. DPPH Radical Scavenging Activity

Equal volumes of peptide solutions (0–992 μM) were mixed with 0.1 mM 2,2-Diphenyl-1-picrylhydrazyl (DPPH) ethanol solution. After a 30 min reaction at room temperature, 200 μL of the reaction mixture was added to a microplate, and absorbance at 517 nm was measured (A_sample_). Controls were set as follows: 200 μL absolute ethanol for 517 nm absorbance (A_blank_) and 200 μL ddH_2_O mixed with 200 μL 0.1 mM DPPH ethanol solution, reacted at room temperature for 30 min. Then, a 200 μL mixture was measured at 517 nm (A_control_).

$$\mathrm{radical}\;\mathrm{scavenging}\;\mathrm{rate}\%=(1-\frac{A_{\mathrm{sample}}-{\;A}_{\mathrm{blank}}}{A_{\mathrm{contral}}})\;\times \;100\%$$

### 2.5. Molecular Dynamics

The three-dimensional structure of the C248 peptide (sequence: CYGRKKRRQRRRALEKEVSVLEKEVSALEKEPGLFYTDKHGEVCPAG) was predicted using the AlphaFold3 online server (https://alphafoldserver.com/, accessed on 4 June 2025). Quantum chemical calculations were carried out with Gaussian 16 software. Density Functional Theory (DFT) in conjunction with the SMD implicit solvation model (scrf = (SMD, solvent = Water)) was employed to simulate an aqueous environment. Geometry optimization was conducted separately for the reactant and product structures. Electronic energies were computed, and thermodynamic corrections were applied to derive the enthalpy change (ΔH) and Gibbs free energy change (ΔG). The prediction of the active pocket in Prdx4 decamers was performed via CASTpFold.

### 2.6. Hydrogen Peroxide Levels

Hydrogen peroxide levels were measured using a Hydrogen Peroxide Assay Kit (Beyotime, Shanghai, China, S0038). For 1 × 10^6^ cells, the kit-provided lysis buffer was added; after ultrasonic disruption, supernatants were collected by centrifugation. Then, 50 μL samples or standards were added to assay wells, followed by 100 μL of detection reagent. After a 30 min incubation at room temperature, the absorbance at 560 nm was measured immediately.

### 2.7. Immunofluorescence Staining

Cells were seeded in confocal dishes and treated for 24 h when confluency reached 60%. After removing the medium, 500 μL of 4% paraformaldehyde was added for 25 min fixation at room temperature. Following 3 PBS rinses, the cells were permeabilized with Beyotime immunostaining permeabilization solution for 10 min and then washed 3 times with PBS (5 min each). After blocking with Biyuntian immunostaining blocking solution for 10 min, primary antibodies (3β-HSD, 1:100, ProteinTech, Rosemont, IL, USA, Cat. No. 15516-1-AP; P21, 1:100, ProteinTech, Cat. No. 28248-1-AP; 4-HNE, 1:25, Abcam, Waltham, MA, USA, Cat. No. ab48506; Mouse IgG, same concentration as the primary antibody, ProteinTech, Cat. No. B900620; Rabbit IgG, same concentration as the primary antibody, ProteinTech, Cat. No. 98136-1-RR) were added and incubated overnight at 4 °C. After removing the primary antibodies and PBS washing, the secondary antibodies were incubated for 1 h (Anti-Mouse secondary antibody, 1:500, Invitrogen, Cat. No. A21202; Anti-Rabbit secondary antibody, 1:500, Invitrogen, Cat. No. A21207). The nuclei were counterstained with Hoechst before mounting (Hoechst 33342, 1:500, Invitrogen, Cat. No. H3570). Fluorescence quantitative analysis was performed using ImageJ version 1.54q (National Institutes of Health, USA), with identical threshold parameters set to measure the fluorescence signal intensity. Cell counting was conducted by enumerating Hoechst-positive cells with intact morphology using ImageJ. At least 150 cells were counted per image. Data were expressed as the total fluorescence intensity of each image divided by the number of cells in the same image.

### 2.8. GSH, CAT, and MDA

After 24 h of drug treatment, the cell pellets in a 6-well plate were collected. The samples were lysed with protein lysis buffer and stored on ice. GSH, catalase, and MDA levels were detected using a reduced glutathione (GSH) assay kit (Nanjing Jiancheng, Nanjing, China, Cat. No. A006-2-1), a Catalase assay kit (Beyotime, Cat. No. S0051), and a Lipid Peroxidation MDA assay kit (Beyotime, Cat. No. S0131S).

For the GSH assay, cell pellets were resuspended in 0.2 mL PBS and ultrasonically disrupted at 4 °C. A 0.1 mL aliquot was mixed with Reagent 1 (precipitant), followed by centrifugation to collect the supernatant. The supernatant was used for BCA protein quantification. For GSH measurement, a blank group (PBS) and a standard group (20 μmol/L GSH standard solution) were included. Briefly, 100 μL of supernatant/PBS/standard was added to each reaction well, followed by 25 μL of buffer and 100 μL of chromogenic solution. The plate was incubated at room temperature for 5 min, and the absorbance of each well was measured at 405 nm using a microplate reader. For the Catalase assay kit, the lysis buffer was used for the test samples.

For CAT detection, 5 μL of a test sample was mixed with 35 μL of catalase assay buffer, followed by the addition of 10 μL of 250 mM hydrogen peroxide solution. The mixture was rapidly blended with a pipette and incubated at room temperature for 3 min; then, the reaction was terminated by adding 450 μL of stop solution. A 10 μL aliquot of the terminated reaction mixture was mixed with 40 μL of buffer, and 10 μL of this dilution was combined with 200 μL of chromogenic working solution. After incubation for 15 min, the absorbance was measured at 520 nm (A520). For the standard curve, serially diluted standard solutions were prepared, and 4 μL of each dilution was mixed with 200 μL of chromogenic working solution for detection under the same conditions.

For the MDA assay, cell pellets were lysed with 200 μL of lysis buffer to prepare test samples, and aliquots were used for BCA protein quantification. For MDA measurement, 0.1 mL of a test sample or serial dilutions of the standard were added to 0.2 mL of the MDA assay working solution. After thorough mixing, the mixture was heated at 100 °C for 15 min, cooled to room temperature, and centrifuged to collect the supernatant. The absorbance of the supernatant was measured at 532 nm (A532) using a microplate reader.

All assays were performed in accordance with the manufacturers’ instructions, and the final concentrations of GSH, CAT, and MDA were normalized to the total protein content (determined by the BCA assay) for quantitative analysis. Three independent biological replicates were conducted for each group.

### 2.9. 8-OHDG

After 24 h of drug treatment, cells were harvested and counted. Approximately 5 × 10^6^ cells were resuspended in 200 μL PBS and subsequently subjected to ultrasonic cell lysis to release intracellular contents. The lysate was then centrifuged at 1500× *g* for 10 min at 4 °C to remove cell debris. Thereafter, the supernatant was collected and used for the detection of 8-hydroxydeoxyguanosine (8-OHDG) following the manufacturer’s instructions for the 8-OHDG ELISA kit (Elabscience, Houston, TX, USA, Cat. No. E-EL-0028).

### 2.10. Cell Viability Assay

Cells were seeded into 96-well plates at a density of 1 × 10^4^ cells per well and treated according to different experimental purposes. After the treatment period, 10 μL of CCK8 reagent (BIOSHARP, Beijing, China, Cat. No. BL502A) was added to each well, and the plates were incubated at 37 °C for 1 h. The absorbance at 450 nm was measured using a microplate reader to evaluate cell viability.

### 2.11. Senescence-Associated β-Galactosidase (SA-β-gal) Staining

The cells were fixed with staining fixative for 10 min and then rinsed twice with PBS to remove residual fixative. Subsequent staining was performed strictly following the manufacturer’s instructions for the SA-β-gal Staining Kit (Beyotime, Cat. No. C0602). After staining, positive cells (characterized by blue staining) were counted under a light microscope, with 5–10 random fields counted per sample to ensure statistical reliability.

### 2.12. RNA Extraction, cDNA Synthesis, Genomic DNA Purification, and qPCR

Total RNA was extracted from the cells using Trizol reagent (Invitrogen, Cat. No. 15596018). The RNA was reverse-transcribed into cDNA using the HiScript III RT SuperMix reverse transcription kit (Vazyme, Nanjing, China, Cat. No. R323—01). qPCR was performed using ChamQ SYBR qPCR Master Mix (Vazyme, Cat. No. Q311—02) on an Applied Biosystems StepOnePlus Real-Time PCR System. The relative expression levels of target genes were calculated using the 2^−ΔΔCt^ method and normalized to β-actin. The primer sequences for Trp53, p21, CYP11A1, 3β-HSD, ND1, and Cox1 are shown in Table 2.

### 2.13. Testosterone Assay

Cell culture supernatants were collected 24 h after treatment and immediately centrifuged at 1000× *g* for 5 to remove any floating cells or debris. The resulting supernatants were either assayed immediately or stored at −80 °C until analysis to prevent testosterone degradation. Testosterone levels were measured strictly following the manufacturer’s instructions for the testosterone ELISA kit (Elabscience, Cat. No. E-EL-M0033c), with all reagents equilibrated to room temperature (25 ± 2 °C) prior to use. After the reaction was completed, absorbance was measured at 450 nm using a microplate reader. Testosterone concentrations were calculated based on a standard curve generated from serial dilutions of testosterone standards, with each sample assayed in duplicate to ensure measurement accuracy.

### 2.14. TUNEL Assay

Cell apoptosis was detected using the TUNEL Brightgreen Apoptosis Detection Kit (Vazyme, Cat. No. A112—01). Briefly, cells were fixed with PFA at room temperature for 20 min and then rinsed three times with PBS. Subsequently, the cells were permeabilized with 0.1% Triton X-100 for 10 min, followed by another three washes with PBS. For the positive control group, the cells were first equilibrated with DNase I Buffer for 5 min and then incubated with 20 U/mL DNase I working solution at room temperature for 10 min. All groups were then treated with Equilibration Buffer for 30 min. The cells were incubated with the freshly prepared TUNEL reaction mixture (negative group without TdT enzyme in the reaction) in a humidified chamber at 37 °C for 60 min, protected from light to prevent fluorophore degradation. Fluorescence signals were visualized using a Leica confocal microscope (STELLARIS) with appropriate excitation/emission filters. For quantification, apoptotic cells (exhibiting positive fluorescent staining) were counted using ImageJ software. Three independent biological replicates were performed, with at least 150 cells counted per replicate.

### 2.15. ROS and Mitochondrial Membrane Potential (ΔΨm) Detection

Intracellular ROS levels were measured using DCFH-DA (Beyotime, Cat. No. S0033), and mitochondrial membrane potential was evaluated using TMRE (Beyotime, Cat. No. C2006). The cells were incubated with 10μM DCFH-DA or 200 nM TMRE at 37 °C for 30 min. After incubation, the cells were washed and analyzed by confocal microscopy using a Leica SP8 STED microscope (Leica Microsystems, Wetzlar, Germany), and the fluorescence intensity was quantitatively measured.

### 2.16. Western Blot Analysis

Total protein was extracted from the cells using RIPA lysis buffer (Beyotime, Cat. No. P0013B), and the protein concentration was determined by a BCA protein quantification kit (Beyotime, Cat. No. P0012S). Thirty micrograms of protein was separated by SDS-PAGE and then transferred onto a PVDF membrane (Millipore, Molsheim, France, Cat. No. ISEQ00010). The membrane was incubated overnight at 4 °C with primary antibodies against p53 (1:10000, proteintech, Cat. No. 80077—1—RR), p21 (1:1000, proteintech, Cat. No. 28248—1—AP), Hsd3b2 (1:5000, proteintech, Cat. No. 15516—1—AP), CYP11A1(1:4000, proteintech, 13363-1-AP), and Vinculin (1:10,000, Abcam, Cat. No. AB129002). After incubation with HRP-conjugated secondary antibodies (1:10,000, proteintech), the protein bands were detected using Super ECL chemiluminescence detection reagent (YEASEN, Shanghai, China, Cat. No. 36208ES76).

### 2.17. Mitochondrial Morphology Analysis

The mitochondria were labeled with 100 nM MitoTracker Deep Red FM (Beyotime, Cat. No. C1046) for 30 min at 37 °C with 5% CO_2_. Meanwhile, the cell nuclei were stained with Hoechst 33342. The cells were imaged using an HIS-SIM Live-Cell Super-Resolution Microscope System (ChaoShiJi Technology, Guangzhou, China). The mean mitochondrial branch length was quantified using the Mitochondria Analyzer plugin in Fiji (ImageJ, National Institutes of Health, USA). Three biological replicates were included per group, with 10 cells analyzed per replicate, resulting in a total of 30 cells for statistical analysis in each group.

### 2.18. ATP Content Assay

The ATP content in the cells was measured according to the instructions for the ATP Assay Kit (Beyotime, Cat. No. S0026). Briefly, cells were lysed using the lysis buffer, and the supernatant was collected after centrifugation (12,000× *g*, 4 °C, 5 min) for subsequent detection. First, 100 μL of ATP assay working solution was added to each test well of a 96-well plate, followed by 20 μL of sample supernatant or ATP standard solution. Luminescence intensity was measured using a multifunctional microplate reader. The ATP concentration of each sample was calculated based on the standard curve, and the results were normalized to the total protein content.

### 2.19. Statistical Analysis

Experimental data were presented as the mean ± standard deviation (mean ± SD) from at least three independent experiments. First, the premise assumptions of one-way analysis of variance (ANOVA) were verified. The data in each group followed a normal distribution, and homogeneity of variances was assessed among groups. If the data were normally distributed and variances were homogeneous, one-way ANOVA followed by Tukey’s post hoc test was performed for multiple comparisons. If variances were heterogeneous, Brown–Forsythe and Welch ANOVA tests were employed instead. Statistical analyses were conducted using GraphPad Prism 10 software (Version 10.1.2). Differences with *p* < 0.05 were considered statistically significant.

## 3. Results

### 3.1. Design of Antioxidant Peptides Based on the Prdx4 Structure

**Figure 1:**
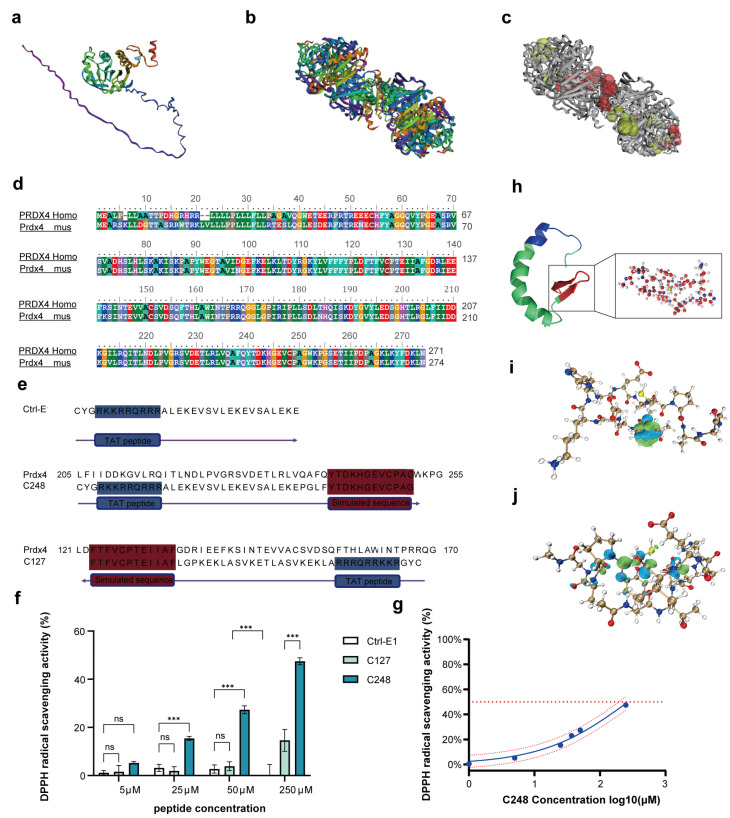
Design of antioxidant peptides based on the Prdx4 structure. (**a**) Cartoon representation of the Prdx4 protein 3D structure. (**b**) Cartoon representation of the 3D structure of Prdx4 polymers. (**c**) Schematic diagram showing the spatial location of the active pocket (highlighted in red) in Prdx4 decamers. This prediction was performed via CASTpFold [27]. (**d**) Alignment of amino acid sequences between human PRDX4 (PRDX4 Homo) and mouse Prdx4 (Prdx4 mus). (**e**) Schematic illustrations of the peptide-negative control sequence (Ctrl-E), C248 sequence, C127 sequence, and Prdx4 amino acid sequence spanning residues 121–170 and 205–255. A TAT peptide (blue box) and a simulated sequence (red box) are indicated in the respective sequences. (**f**) Quantitative analysis of DPPH free radical scavenging activity of different peptides at different concentrations; *n* = 3 samples per group. (**g**) Dose–response relationship between concentrations of C248 and DPPH free radical scavenging activity. The red doted lines flanking the blue line indicate the range of the 95% confidence interval (95% CI) for the values. *n* = 3 samples per group. (**h**) Cartoon representation of the C248 3D structure; the red region indicates the core antioxidant sequence (YTDKHGEVCPAG), the blue region indicates TAT sequence. (**i**) The map of HOMO electron density distribution. The blue/green lobes correspond to the electron distribution regions of the HOMO (highest unoccupied molecular orbital). (**j**) The map of LUMO electron density distribution, The blue/green lobes correspond to the electron distribution regions of the LUMO (lowest unoccupied molecular orbital). ^ns^ *p* > 0.05, *** *p* < 0.0001.

To investigate the antioxidant active sites of the Prdx4 protein, we predicted the three-dimensional (3D) structure of the Prdx4 monomer using AlphaFold (Figure 1a). Subsequently, we employed the CASTp tool to predict active pockets in the 3D structure of the Prdx4 multimeric assembly (Figure 1b,c), and screened the top 10 potential active pockets based on parameters, including pocket volume and accessible surface area (Table 3). Combined with UniProt annotation analysis, the results revealed that the active pockets harboring amino acid residues at positions 127 and 248 of Prdx4 accounted for half of these top 10 key pockets. This distribution pattern suggested that residues 127 and 248 may represent core regions through which Prdx4 mediates substrate binding, catalytic reactions, or dynamic conformational regulation. Furthermore, the comparative analysis demonstrated that human Prdx4 and mouse Prdx4 share identical sequences in the fragment region containing the antioxidant active sites (Figure 1d).

Guided by the identified active sites, we engineered peptides C127 and C248 with an N-terminal TAT cell-penetrating sequence for membrane translocation, alongside a negative control peptide E1-ctrl (Figure 1e). To evaluate their antioxidant capacity, a DPPH radical scavenging assay was conducted. Among the three peptides, C248 showed a significantly higher scavenging activity against DPPH radicals (Figure 1f,g), with an EC50 value of 268.4 μM (*p* < 0.05) (Table 4). To further elucidate the ROS-scavenging capacity of C248, a detailed analysis of its electronic properties was conducted. The three-dimensional structure of C248 was predicted using AlphaFold3 (Figure 1h). The per-residue confidence score (pLDDT) indicated that 87% of the residues were of high confidence (pLDDT ≥ 70). This finding indicates that C248 possesses excellent structural stability and high predictive reliability, which supports the feasibility of subsequent functional investigations. To characterize the reactivity of C248 toward H_2_O_2_, we performed quantum chemical calculations using highest occupied molecular orbital (HOMO) and lowest unoccupied molecular orbital (LUMO) analyses. The HOMO electron density distribution highlighted electron-donating regions (Figure 1i), whereas LUMO indicated electron-accepting sites (Figure 1j). Single-point energy calculations revealed a standard molar Gibbs free energy of formation (ΔG) of −4926.208196 Hartree for the active fragment of C248 and −151.527532 Hartree for H_2_O_2_. Following thermodynamic correction, the reaction enthalpy (ΔH) and Gibbs free energy (ΔG) were determined to be −282.0968475 kJ/mol and −249.2623445 kJ/mol, respectively (Table 5). These results confirm that the reaction is both exothermic and spontaneous.

### 3.2. C248 Ameliorates H_2_O_2_-Induced Senescence in LCs

Low concentrations of H_2_O_2_ can induce the senescence of LCs. Previous studies have confirmed that H_2_O_2_ successfully induces the senescence of embryonic fibroblasts [28]. Our experiments showed that H_2_O_2_ stimulation can dose-dependently decrease LC viability (Figure S1a–d). As the H_2_O_2_ concentration increased in the medium, the proliferation rate of LCs slowed, and the cells exhibited an elongated morphological change. When the H_2_O_2_ concentration reached 400 μM or higher in the medium, the LCs displayed additional morphological features of excessive damage, such as shrinkage and fragmentation (Figure S1e). Concurrently, the proportion of senescence-associated β-galactosidase (SA-β-gal)-positive cells increased in a dose-dependent manner (Figure S1f,g), and the mRNA and protein levels of the senescence-related factors Trp53 and p21 were significantly upregulated (Figure S1h–j).

**Figure 2:**
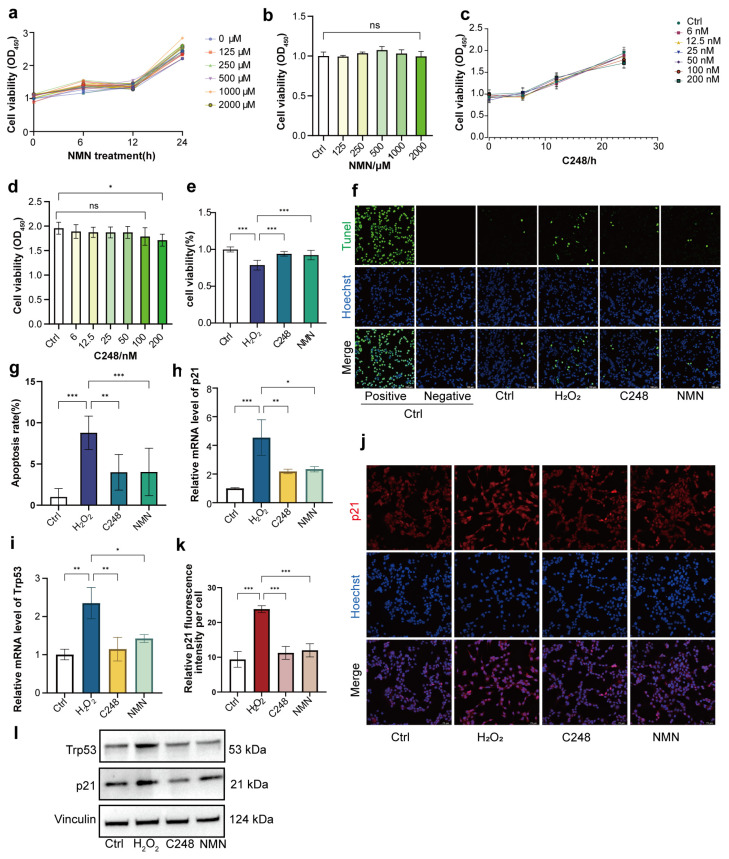
C248 ameliorates H_2_O_2_-induced senescence in LCs. (**a**) Optical density value of MLTC-1 cells determined by a CCK-8 assay. Cells treated with NMN (0, 125, 250, 500, 1000, 2000 µM) for 0, 12, 24, or 48 h. *n* = 5 wells per group. (**b**) Cell viability of MLTC-1 treated with NMN for 24 h. *n* = 5 wells per group. (**c**) Optical density value of MLTC-1 cells determined by a CCK-8 assay. Cells treated with C248 (0, 6, 12.5, 25, 50, 100, 200 nM) for 0, 6, 12, or 24 h. *n* = wells 5 per group. (**d**) Cell viability of MLTC-1 treated with C248 for 24 h. *n* = 5 wells per group. (**e**) Cell viability of MLTC-1 treated with the indicated drugs for 24 h. Ctrl: no treatment; H_2_O_2_: 100 µM H_2_O_2_ treatment; C248: 100 µM H_2_O_2_ + 50 nM C248 treatment. *n* = 5 wells per group. (**f**) Representative images of TUNEL staining in MLTC-1 cells treated with H_2_O_2_/H_2_O_2_ + C248/H_2_O_2_ + NMN for 24 h. Scale bar: 100 µm. (**g**) Quantitative analysis of the TUNEL-positive rate. *n* = 3 samples per group. (**h**,**i**) Quantitative RT-PCR analysis of senescence markers (p21, Trp53) in the indicated groups. *n* = 3 samples per group. (**j**) Representative images of p21 staining in MLTC-1 cells treated with H_2_O_2_/H_2_O_2_ + C248/H_2_O_2_ + NMN for 24 h. Scale bar: 75 µm. (**k**) Quantitative analysis of fluorescence intensity. *n* = 3 samples per group. (**l**) Representative Western blots for Trp53, p21, and vinculin. *n* = 3 samples per group. ^ns^ *p* > 0.05, * *p* < 0.05, ** *p* < 0.01, and *** *p* < 0.0001.

To investigate whether C248 could alleviate the H_2_O_2_-induced senescence of LCs, we first assessed the cytotoxicity of C248 on LCs (NMN as the control). The results showed that within the concentration range tested in this experiment, NMN had no significant effect on cell viability. Similarly, no obvious cytotoxicity was observed for C248 when its concentration was ≤100 nM (Figure 2a–d). Subsequently, the senescent cells were treated with 50 nM C248 or 500 μM NMN, respectively, and both C248 and NMN significantly improved the viability of senescent cells (Figure 2e). To evaluate the effect of C248 on apoptosis in the senescent cells, TUNEL staining was performed to label DNA fragmentation. The senescent cells treated with H_2_O_2_ exhibited a significantly higher percentage of TUNEL-positive cells (8.8 ± 2.0%, *p* ≤ 0.05) compared with the non-senescent control cells (1.0 ± 0.6%). Notably, the treatment with C248 or NMN markedly reduced the proportion of apoptotic cells (Figure 2f,g). Quantitative real-time polymerase chain reaction (qRT-PCR) analysis revealed that the supplementation with C248 or NMN significantly downregulated the mRNA expression levels of the senescence markers p21 and Trp53 (Figure 2h,i). Further immunofluorescence staining analysis showed that the C248 and NMN treatments significantly decreased the protein expression level of p21 in the senescent cells (Figure 2j,k and Figure S4a). Western blot quantification analysis confirmed that C248 or NMN significantly reduced the expression levels of the p21 and Trp53 proteins (Figure 2l). Collectively, these results demonstrate that both C248 and NMN effectively alleviate the senescence of LCs.

### 3.3. C248 Improves Androgen Biosynthesis in Aging LCs

Cellular senescence is often accompanied by a decline in core cellular functions. For LCs, their key physiological function is androgen biosynthesis. In the present study, we found that in the H_2_O_2_-induced senescence model of LCs, the expression levels of two key enzymes in androgen biosynthesis, CYP11A1 and 3β-HSD, were significantly decreased at both the transcriptional level and the protein level (Figure S2b–d). Concurrently, the amount of testosterone secreted also decreased (Figure S2a). These findings further confirm that H_2_O_2_-induced senescence impairs the androgen biosynthetic function of LCs.

**Figure 3:**
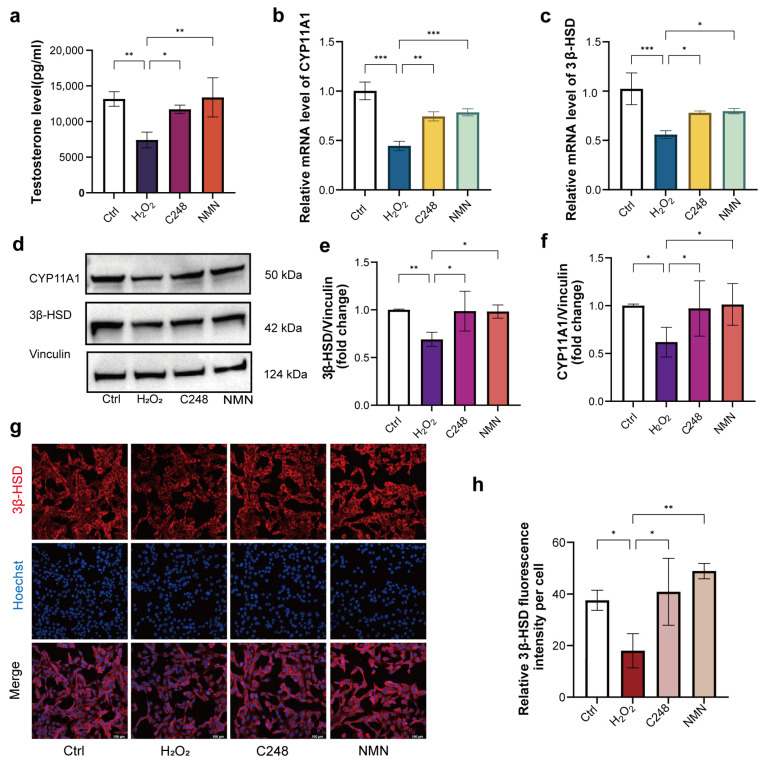
C248 improves androgen biosynthesis in aging LCs. (**a**) Testosterone production of MLTC-1 cell culture. *n* = 3 samples per group. (**b**,**c**) Quantitative RT-PCR analysis of CYP11A1 and 3β-HSD in the indicated groups. *n* = 3 samples per group. (**d**) Representative Western blots for CYP11A1,3β-HSD and vinculin. (**e**,**f**) Quantitative analysis of CYP11A1 and 3β-HSD protein levels relative to vinculin. Cells were treated with H_2_O_2_/H_2_O_2_ + C248/H_2_O_2_ + NMN for 24 h. *n* = 3 samples per group. (**g**) Representative images of 3β-HSD staining in MLTC-1 cells treated with H_2_O_2_/H_2_O_2_ + C248/H_2_O_2_ + NMN for 24 h. Scale bar: 100 µm. (**h**) Quantitative analysis of relative fluorescence intensity. *n* = 3 samples per group. * *p* < 0.05, ** *p* < 0.01, and *** *p* < 0.0001.

To determine whether C248 can reverse the aforementioned functional impairment, we investigated the effects of C248 on androgen biosynthesis and secretion in the senescent LCs. First, the senescent cells were treated with C248 for 24 h (NMN as the control). Subsequently, the cell culture medium was collected, and the testosterone concentration in the medium was measured using an enzyme-linked immunosorbent assay (ELISA). The results showed that both C248 and NMN effectively restored the androgen-secreting capacity of senescent cells (Figure 3a). Given that androgen biosynthesis involves multiple enzymatic reactions, and CYP11A1 and 3β-HSD are two key rate-limiting enzymes in the steroidogenesis pathway, we further detected the expression levels of CYP11A1 and 3β-HSD. The results demonstrated that the supplementation with C248 or NMN significantly upregulated the mRNA and protein expression levels of CYP11A1 and 3β-HSD in the senescent cells (Figure 3b–f). Furthermore, the protein expression level of 3β-HSD was further verified by immunofluorescence staining. The result was consistent with those of the Western blot and qRT-PCR, confirming that C248 or NMN could effectively restore the protein expression of 3β-HSD in senescent cells (Figure 3g,h and Figure S4b).

### 3.4. The Ameliorative Effects of C248 Related to Antioxidation

**Figure 4:**
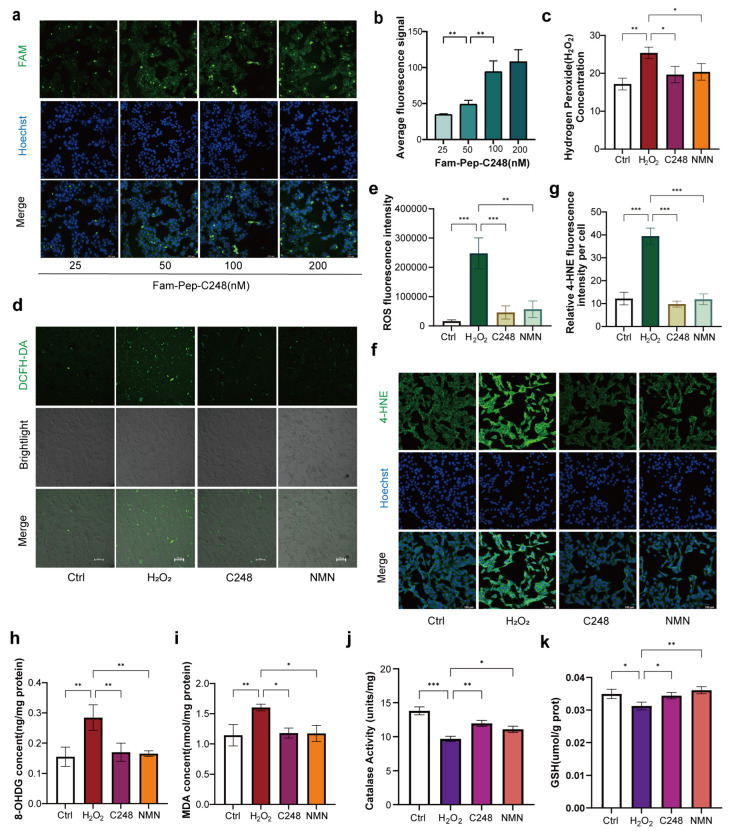
The ameliorative effects of C248 related to antioxidation. (**a**) Representative images of FAM staining in MLTC-1 cells treated with C248 at different concentration (25, 50, 100, 200 nM) for 4 h. C248 was conjugated with the FAM fluorescent tag. (**b**) Quantitative analysis of relative fluorescence intensity. *n* = 3 samples per group. (**c**) Hydrogen peroxide (H_2_O_2_) concentration in MLTC-1 cells treated with H_2_O_2_/H_2_O_2_ + C248/H_2_O_2_ + NMN for 24 h. *n* = 3 samples per group. (**d**) Representative images of DCFH-DA staining in MLTC-1 cells treated with H_2_O_2_/H_2_O_2_ + C248/H_2_O_2_ + NMN for 6 h. (**e**) Quantitative ROS analysis of relative DCFH-DA fluorescence intensity. *n* = 3 samples per group. (**f**) Representative images of 4-HNE staining in MLTC-1 cells treated with H_2_O_2_/H_2_O_2_ + C248/H_2_O_2_ + NMN for 24 h. (**g**) Quantitative analysis of relative 4-HNE fluorescence intensity. *n* = 3 samples per group. (**h**,**i**) 8-OHDG and MDA levels in cells. *n* = 3 samples per group. (**j**,**k**) Relative analysis of enzyme activity of Catalase and GSH. *n* = 3 samples per group. * *p* < 0.05, ** *p* < 0.01, and *** *p* < 0.0001.

Given that C248 can directly scavenge ROS, we further investigated its role in consuming intracellular ROS in LCs. Firstly, to confirm whether C248 can enter into LCs, we conjugated a FAM fluorescent tag to the C-terminal of the C248 molecule. After co-incubating the tagged C248 with LCs for 4 h, fluorescent signals were detected in the cells. The results showed a dose-dependent increase in intracellular fluorescence intensity (Figure 4a,b and Figure S5a,b). This indicated that C248 can smoothly penetrate the cell membrane to enter the cytoplasm and thereby exert its synergistic effects, both inside and outside of the cell. Subsequently, we examined the effects of C248 on the intracellular H_2_O_2_ level (NMN as the control). The results demonstrated that both the C248 and NMN treatments significantly reduced the intracellular H_2_O_2_ content in the senescent cells (Figure 4c). Further investigation into the short-term effect of C248 revealed that C248 rapidly and effectively decreased the total intracellular ROS levels in the LCs (Figure 4d,e). Treatment with C248 led to a dose-dependent decrease in intracellular ROS levels, suggesting that C248 plays a key role in mediating the reduction in intracellular ROS (Figure S3a,b). To comprehensively evaluate the ameliorative effect of C248 on oxidative damage, we further detected the levels of key peroxidation products in the senescent cells, including the lipid peroxidation products 4-hydroxynonenal (4-HNE) and malondialdehyde (MDA), as well as the DNA oxidative damage marker 8-hydroxy-2′-deoxyguanosine (8-OHDG). The results showed that the C248 and NMN treatments significantly downregulated the levels of these three peroxidation products (Figure 4f–i and Figure S4c). The antioxidant enzyme system is a core defense mechanism against oxidative stress. We further examined the effect of C248 on key indicators of this system. The results showed that the C248 treatment effectively maintained the stable level of glutathione (GSH) in the senescent cells and significantly increased the activity of catalase (CAT) (Figure 4j,k).

### 3.5. C248 Improves Mitochondrial Function of LCs

**Figure 5:**
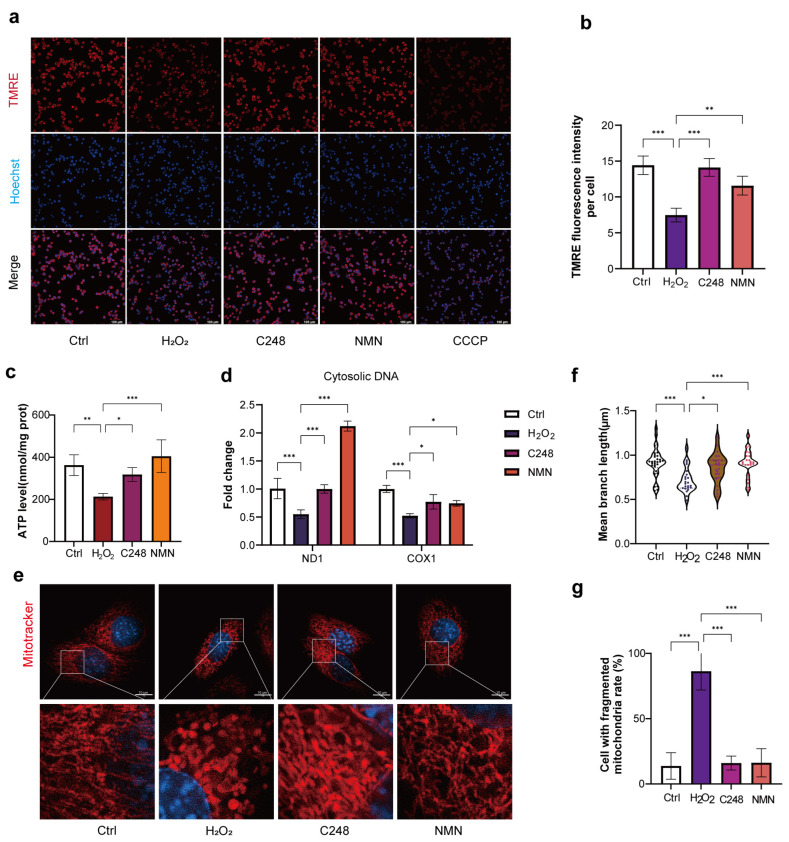
C248 improves the mitochondrial function of LCs. (**a**) Representative images of TMRE staining in MLTC-1 cells treated with H_2_O_2_/H_2_O_2_ + C248/H_2_O_2_ + NMN for 6 h. (**b**) Quantitative analysis of TMRE relative fluorescence intensity. *n* = 5 samples per group. (**c**) ATP level in MLTC-1 cells treated with H_2_O_2_/H_2_O_2_ + C248/H_2_O_2_ + NMN for 24 h. *n* = 3 samples per group. (**d**) Quantification of cytosolic mtDNA (ND1, COX1) in MLTC-1 cells treated with H_2_O_2_/H_2_O_2_ + C248/H_2_O_2_ + NMN for 24 h. *n* = 3 samples per group. (**e**) Mitochondrial performance in MLTC-1 cells treated with H_2_O_2_/H_2_O_2_ + C248/H_2_O_2_ + NMN for 4 h. Cells were labeled for the mitochondrial inner membrane using Mitotracker Deep Red FM. *n* = 3 samples per group and 10 cell images per sample. (**f**) Mean branch length in MLTC-1 cells. The colorful dots correspond to the raw measurement data points of each individual sample in the respective group. (**g**) Quantification of a cell with fragmented mitochondria. * *p* < 0.05, ** *p* < 0.01, and *** *p* <0.001.

Given that oxidative stress triggers mitochondrial functional impairment, we further investigated the effects of peptide C248 on the restoration of mitochondrial function in LCs (NMN as the control). First, a TMRE fluorescent probe was used to detect mitochondrial membrane potential. The results showed that both the C248 and NMN treatments significantly reversed the oxidative stress-induced decrease in mitochondrial membrane potential (Figure 5a,b), suggesting that both molecules can maintain the homeostasis of basic mitochondrial functions. ATP is a key indicator of mitochondrial energy metabolism, and its level is directly related to the mitochondrial functional status. In the present study, our results demonstrated that the C248 and NMN treatments effectively increased the ATP level in the senescent cells (Figure 5c), indicating that both molecules can enhance mitochondrial energy production capacity.

MtDNA copy number decreases with the progression of cellular senescence and oxidative damage. Notably, the ND1 and COX1 gene fragments in mtDNA are highly conserved, and their copy numbers can indirectly reflect the quantity and functional status of mitochondria. In the present study, we further detected the copy numbers of these two genes. The results showed that both peptide C248 and NMN could effectively maintain the mtDNA copy number in the senescent LCs (Figure 5d), demonstrating that both molecules can protect mtDNA from oxidative damage.

Finally, to evaluate changes in mitochondrial morphology, we used a Mitotracker fluorescent probe to specifically label the mitochondrial membrane of living cells. Combined with high-resolution structured illumination microscopy (HIS-SIM), we observed the proportion of cells with abnormal mitochondrial morphology. Our results showed that C248 alleviated mitochondrial fragmentation and preserved the tubular structure of mitochondria (Figure 5e–g). Collectively, these results demonstrate that C248 can improve mitochondrial function in Leydig cells.

## 4. Discussion

It is more and more important to explore the prevention of testicular senescence and the therapeutic strategy of LOH in medium-elderly men [29]. Given that oxidative stress is a pivotal driver of cell senescence, the vicious cycle between oxidative stress and mitochondrial dysfunction should be a target [30]. Besides some antioxidants, melatonin has been utilized to ameliorate mitochondrial dysfunction and restore physiological function in the context of age-related neurodegeneration [31]. In the present study, we first designed an antioxidant peptide, C248, based on the molecular structure of PRDX4. Utilizing radical scavenging assays and molecular dynamics simulations, we observed that it possesses the property of scavenging oxygen free radicals. We further evaluated its effects on Leydig cells for a future application in the prevention of testicular senescence and the treatment of LOH. Our results showed that the bioactive peptide C248 of PRDX4 and NMN could significantly improve the mitochondrial function of LCs, and that peptide C248 alleviated LC senescence and improved androgen biosynthesis in LCs.

At present, some antioxidants have been investigated as adjunctive treatments to improve the testicular function of medium-elderly men in clinical practice. The combined use of antioxidants, like L-carnitine and coenzyme Q, is thought to improve sperm quality [32,33]. In recent years, a promising research direction has investigated bioactive small molecules with the potential for antioxidation and functional protection of testicular LCs by delaying senescence. With the optimization of functional peptide synthesis technology, the research of bioactive peptides has made significant progress in multiple fields. Some antioxidant peptides from plants with known sequences can be chemically synthesized, and they have shown good antioxidant capacity in cell models [21]. Studies have found that antimicrobial peptides screened by AI can target and penetrate deep into cell membranes to exert antimicrobial effects [34]. It has been reported that Liraglutide, as another bioactive peptide, can improve mitochondrial performance and reduce oxidative stress in mouse LCs [35]. Previous studies have identified Peroxiredoxin 4 (PRDX4) as a critical antioxidant enzyme in the reproductive system [26]. A defining feature of PRDX4 is the presence of two highly conserved cysteine (Cys) residues at positions 127 and 248 of its peptide chain, which are recognized as two key functional sites underlying the biological activity [26]. Through active-site prediction analyses of the Prdx4 protein, we also identified that the active pockets harboring the Cys127 and Cys248 residues serve as critical regions for mediating the redox reaction. The N-terminal segment of each peptide incorporates three amino acids (CYG) and the TAT cell-penetrating peptide (CPP) sequence. The middle segment consists of the structural linker “ALEKEVSVLEKEVSALEKEPGLF,” which stabilizes the peptide conformation. This rigid amphipathic α-helix serves as a scaffold to connect functional domains, preventing disordered peptide folding. Additionally, hydrophobic interactions between hydrophobic amino acids within this helix confer resistance to intracellular protease degradation [36]. The fourth segment comprises the core antioxidant mimetic sequence, either “AIIETPCVFTF” (for C127) or “YTDKHGEVCPAG” (for C248). Our results revealed that C248 exhibited superior free radical scavenging capacity and could directly react with H_2_O_2_. Collectively, these findings strongly demonstrate that peptide C248 is a promising candidate molecule with potential antioxidant activity.

Our findings revealed that peptide C248 can downregulate the expression of senescence-associated factors in LCs at both the mRNA and protein levels. Testicular senescence refers to the decline in testicular function, and the dysfunction of LCs contributes to the decline in androgen levels, which is closely associated with LOH [5]. Similarly, in the H_2_O_2_-induced senescence model of LCs, we observed the impaired function of androgen biosynthesis and secretion. Some peptide drugs have been proposed to treat senescence-associated diseases in clinical practice, such as GLP-1 class drugs and SS-31 [37,38]. In the present study, the design strategy for two bioactive peptides of C248 and C127, as antioxidants based on the PRDX4 protein structure, can provide a new idea for other studies on antioxidants and mimetic ligands. Our study further corroborates the role of peptide C248 in restoring the physiological function of senescent LCs.

Peptide C248 showed a pivotal role in alleviating oxidative stress levels in the senescent LCs in this study. When the cells are exposed to excessive ROS, intracellular oxidative stress is induced, which is characterized by elevated levels of peroxidation products and dysregulation of the antioxidant enzyme system [39]. We found that peptide C248, with NMN as the positive control, can reduce the peroxidation products of lipids and DNA and enhance the activity and expression of antioxidant enzymes. Notably, ROS are closely intertwined with mitochondria. Mitochondria themselves generate ROS during biosynthetic processes, while excessive ROS directly targets mitochondria [12]. Mitochondria govern energy metabolism and biosynthesis in LCs, and mitochondrial quality to a certain extent reflects the functional status of these cells. Our results demonstrated that both C248 and NMN reduced the intracellular ROS level. Furthermore, we found that peptide C248 restored the level of mitochondrial ATP production, maintained the mitochondrial DNA (mtDNA) copy number, and preserved the mitochondrial morphology in LCs. These results indicate that peptide C248 can feasibly improve the function of senescent LCs by enhancing mitochondrial function. However, the stability of biological activity, efficacy, and safety of peptide C248 as an antioxidant requires further research, and exploration based on animal models will be necessary in the future.

## 5. Conclusions

In summary, our results have demonstrated that peptide C248 of PRDX4 alleviates oxidative stress-induced LC senescence via distinct but complementary mechanisms. Peptide C248 mitigates LC senescence by scavenging free radicals and reducing mitochondrial oxidative damage, while NMN acts by restoring mitochondrial energy metabolism. Both peptide C248 and NMN converge on mitochondrial protection to improve LC viability and testosterone secretion. These findings identify peptide C248 of PRDX4 as a novel candidate antioxidant and a potential activator of LC mitochondria, suggesting a viable strategy for the treatment of LOH and delaying the progress of LOH.

## Figures and Tables

**Table 1:** Sequence information of peptides.

Name	Sequence	Molecular Weight	Purity
Pep-Ctrl	CYGRKKRRQRRRALEKEVSVLEKEVSALEKE	3775.4	>98%
Pep-C248	CYGRKKRRQRRRALEKEVSVLEKEVSALEKEPGLFYTDKHGEVCPAG	5448.28	
Pep-C127	CYGRKKRRQRRRALKEKVSALTEKVSALEKEPGLFAIIETPCVFTF	5354.43	

**Table 2:** Primer sequence information.

Primer Name	Primer Sequence	Gene Accession
Trp53	F: TCCGAAGACTGGATGACTGC R: GATCGTCCATGCAGTGAGGT	AF074575
p21	F: TCGCTGTCTTGCACTCTGGTGT R: CCAATCTGCGCTTGGAGTGATAG	AC167363
CYP11A1	F: TGCTCAACCTGCCTCCAGACTT R: ACTGGCTGAAGTCTCGCTTCTG	AC158996
3β-HSD	F: ATCAGGGTCCTGGACAAGGTCT R: TGGCAAGCTCTCCTCAGGTACT	AL606755
ND1	F: CCCTAAAACCCGCCACATCT R: GAGCGATGGTGAGAGCTAAGGT	DQ874614
Cox1	F: GCCCCAGATATAGCATTCCC R:GTTCATCCTGTTCCTGCTCC	DQ874614
β-actin	F: CATTGCTGACAGGATGCAGAAGG R: TGCTGGAAGGTGGACAGTGAGG	AC144818

**Table 3:** CASTp-predicted top 10 active sites of PRDX4.

Pocket ID	Area (SA) (Å^2^)	Volume (SA) (Å^3^)	Annotation	Position
1	727.919	712.504	-	-
2	422.483	495.469	Active site, disulfide bond	127, 248
3	301.884	235.534	Active site, disulfide bond	127, 248
4	320.175	214.059	-	-
5	284.450	205.076	-	-
6	244.699	203.740	Active site, disulfide bond	127, 248
7	282.439	191.241	-	-
8	201.537	141.881	Active site, disulfide bond	127, 248
9	162.097	116.080	Active site, disulfide bond	127, 248
10	223.853	114.251	-	-

The data were calculated via the CASTpFold website [27].

**Table 4:** The DPPH radical scavenging activity of C248.

	EC50	Confidence Interval
C248	268.4 µM	[230.5, 319.7]

**Table 5:** Molecular energy data from HOMO and LUMO calculations for the reaction.

		Standard Molar Enthalpy of Formation (Hartree)	Standard Molar Free Energy of Formation (Hartree)
Reactants	peptide-SH	−4926.208196	−4926.449746
	H_2_O_2_	−151.527532	−151.553393
Products	peptide-SOH	−5001.435886	−5001.669346
	H_2_O	−76.407287	−76.428732
Enthalpy changes (ΔH)		−0.107445	-
The Gibbs free energy change (ΔG)		-	−0.094939

## Data Availability

The original contributions presented in this study are included in the article/Supplementary Material. Further inquiries can be directed to the corresponding author.

## Supplementary Materials

The following supporting information can be downloaded at https://www.mdpi.com/article/10.3390/antiox15010021/s1: Figure S1: senescence in LCs induced by H_2_O_2_. (a) Optical Density value of MLTC-1 cells determined by the CCK-8 assay. Cells treated with H_2_O_2_ (0, 25, 50, 100, 200, 400, 500, 800, 1000, 2000 µM) for 0, 6, 12 or 24 h. *n* = 5 wells per group. (b–d) Cell viability of MLTC-1 treated with H_2_O_2_ (0, 25, 50, 100, 200, 400, 500, 800, 1000, 2000 µM) for 6, 12 or 24 h. *n* = 5 wells per group. (e) Cell performance of MLTC-1 treated with H_2_O_2_ (50, 100, 200, 400, 500, 800 µM) after culture for 12 and 24 h. Scale bar: 200 µm. *n* = 3 samples per group. (f) Representative images of SA-β-gal staining in MLTC-1 cells of the indicated groups. Senescent cells were treated with H_2_O_2_ for 24 h before staining. Scale bar: 100 µm. (g) Quantitative analysis of SA-β-gal+ cells. *n* = 3 samples per group. (h,i) Quantitative RT-PCR analysis of senescence markers(p21, Trp53) of MLTC-1 treated with H_2_O_2_ (50, 100, 200, 400, 800µM) for 24 h. *n* = 3 samples per group. (j) Representative western blots for Trp53, p21 and vinculin. *n* = 3 samples per group. ^ns^ *p* > 0.05, ** *p* < 0.01, *** *p* < 0.0001. Figure S2: Senecent LCs with impaired androgen biosynthesis. (a) Testosterone production of MLTC-1 cell culture treated with H_2_O_2_ (50, 100, 200, 400, 800µM) for 24 h. *n* = 3 samples per group. (b,c) Quantitative RT-PCR analysis of CYP11A1 and 3β-HSD in the indicated groups. *n* = 3 samples per group. (d) Representative western blots for CYP11A1,3β-HSD and vinculin. *n* = 3 samples per group. * *p* < 0.01, ** *p* < 0.01, *** *p* < 0.0001. Figure S3: ROS level of LCs treated with C248. (a) Representative images of DCFH-DA staining in MLTC-1 cells treated with H_2_O_2_/H_2_O_2_ + C248 (5nM, 50nM, 100nM, 200nM) for 6h. All images were taken with a confocal laser scanning microscope (Leica STELLARIS5). *n* = 3 samples per group. (b) Quantitative ROS analysis of relative DCFH-DA fluorescence intensity. *n* = 3 samples per group. ^ns^ *p* > 0.05 * *p* < 0.01, ** *p* < 0.01, *** *p* < 0.0001. Figure S4: Negative control in immunofluorescence. (a) Representative images of IgG rabbit staining in MLTC-1 cells(negative control of P21 staining) (b) Representative images of IgG rabbit staining in MLTC-1 cells(negative control of 3β-HSD staining). (c)Representative images of IgG muose staining in MLTC-1 cells(negative control of 4-HNE staining). Figure S5: Effect of Fixation on FAM Fluorescence. (a) Representative images of FAM staining in MLTC-1 cells treated with low or high concentration about C248 and fixed or unfixed samples. (b) Quantitative analysis of relative FAM fluorescence intensity. *n* = 3 samples per group. * *p* < 0.01, ** *p* < 0.01, *** *p* < 0.0001.
