# Supplementary material for: Bioactive Peptide C248 of PRDX4 Ameliorates the Function of Testicular Leydig Cells via Mitochondrial Protection
Source: Antioxidants (Basel). 2025 Dec 22;15(1):21. doi: 10.3390/antiox15010021 (PMC12837840; doi:10.3390/antiox15010021)
Supplement: Supplementary file 1 [file antioxidants-15-00021-s001.zip › antioxidants-3987790-supplementary.pdf]

Supplementary Figure

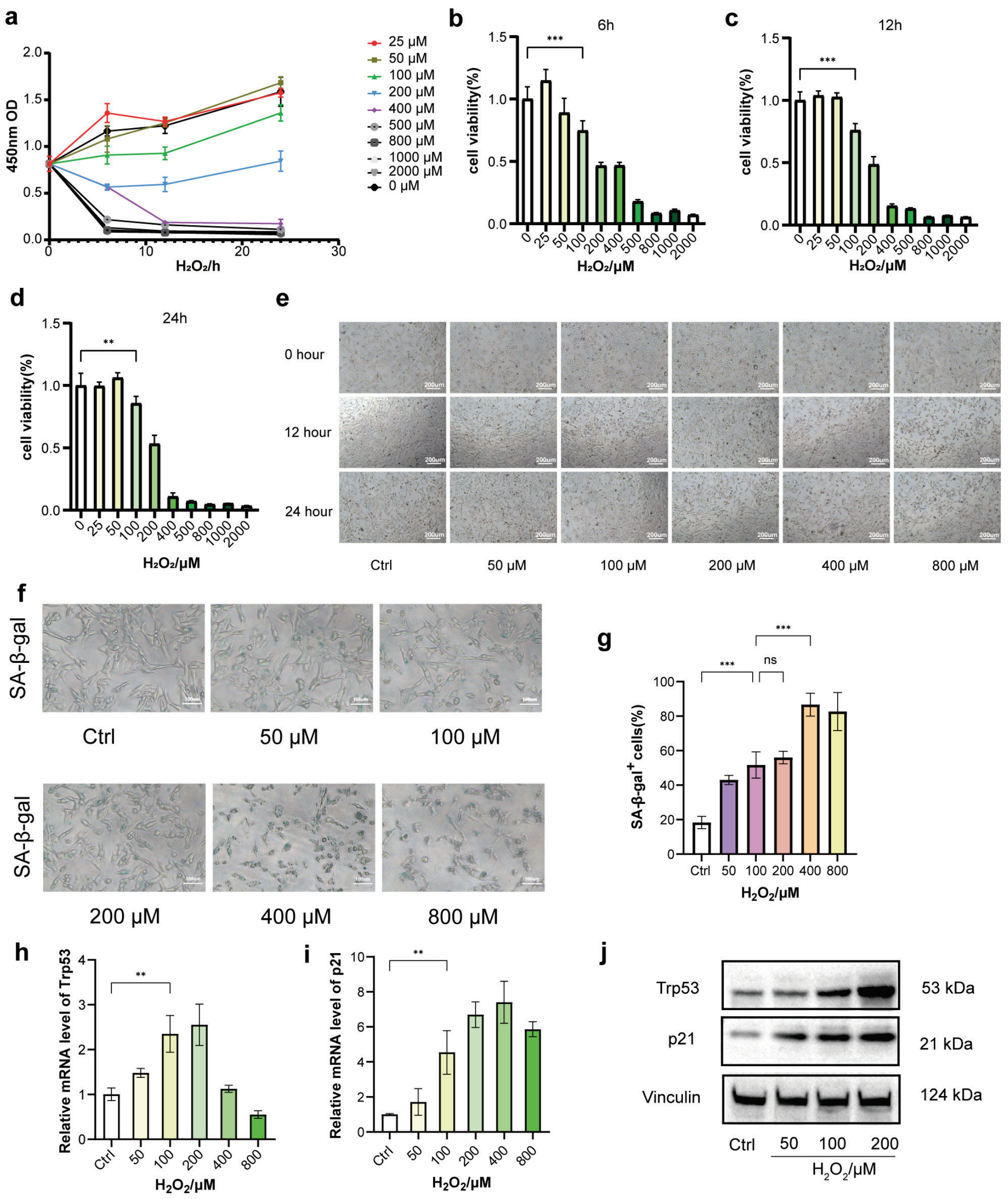

Figure S1. senescence in LCs induced by H_2O_2. (a) Optical Density value of MLTC-1 cells determined by the CCK-8 assay. Cells treated with H_2O_2（0,25, 50, 100,200,400, 500, 800, 1000,2000μM）for 0, 6, 12 or 24 hours. n=5 wells per group. (b-d) Cell viability of MLTC-1 treated with H_2O_2（0,25, 50, 100,200,400, 500, 800, 1000,2000μM）for 6, 12 or 24 hours. n=5 wells per group. (e) Cell performance of MLTC-1 treated with H_2O_2（50, 100,200,400, 500, 800μM）after culture for 12 and 24hours. Scale bar: 200 μm. n=3 samples per group. (f) Representative images of SA-β-gal staining in MLTC-1 cells of the indicated groups. Senescent cells were treated with H_2O_2 for 24 hours before staining. Scale bar: 100 μm. (g) Quantitative analysis of SA-β-gal+ cells. n=3 samples per group. (h-i) Quantitative RT-PCR analysis of senescence markers(p21, Trp53) of MLTC-1 treated with H_2O_2（50, 100,200,400, 800μM）for 24 hours. n=3

samples per group. (j) Representative western blots for Trp53, p21 and vinculin. n=3 samples per group. *p<0.05, **p<0.01, ***p<0.0001.

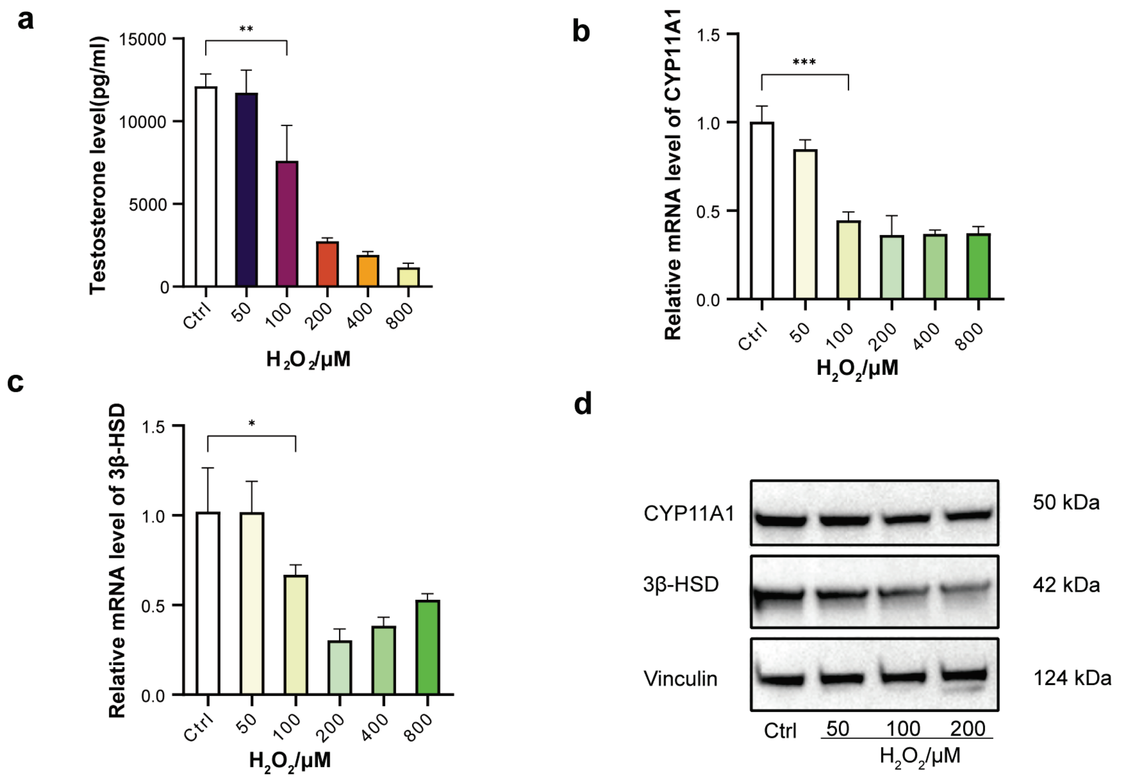

Figure S2. Senecent LCs with impaired androgen biosynthesis. (a)Testosterone production of MLTC-1 cell culture treated with H_2O_2（50, 100, 200, 400, 800μM） for 24 hours. n=3 samples per group. (b-c) Quantitative RT-PCR analysis of CYP11A1 and 3β-HSD in the indicated groups. n=3 samples per group. (d) Representative western blots for CYP11A1,3β-HSD and vinculin. n=3 samples per group.

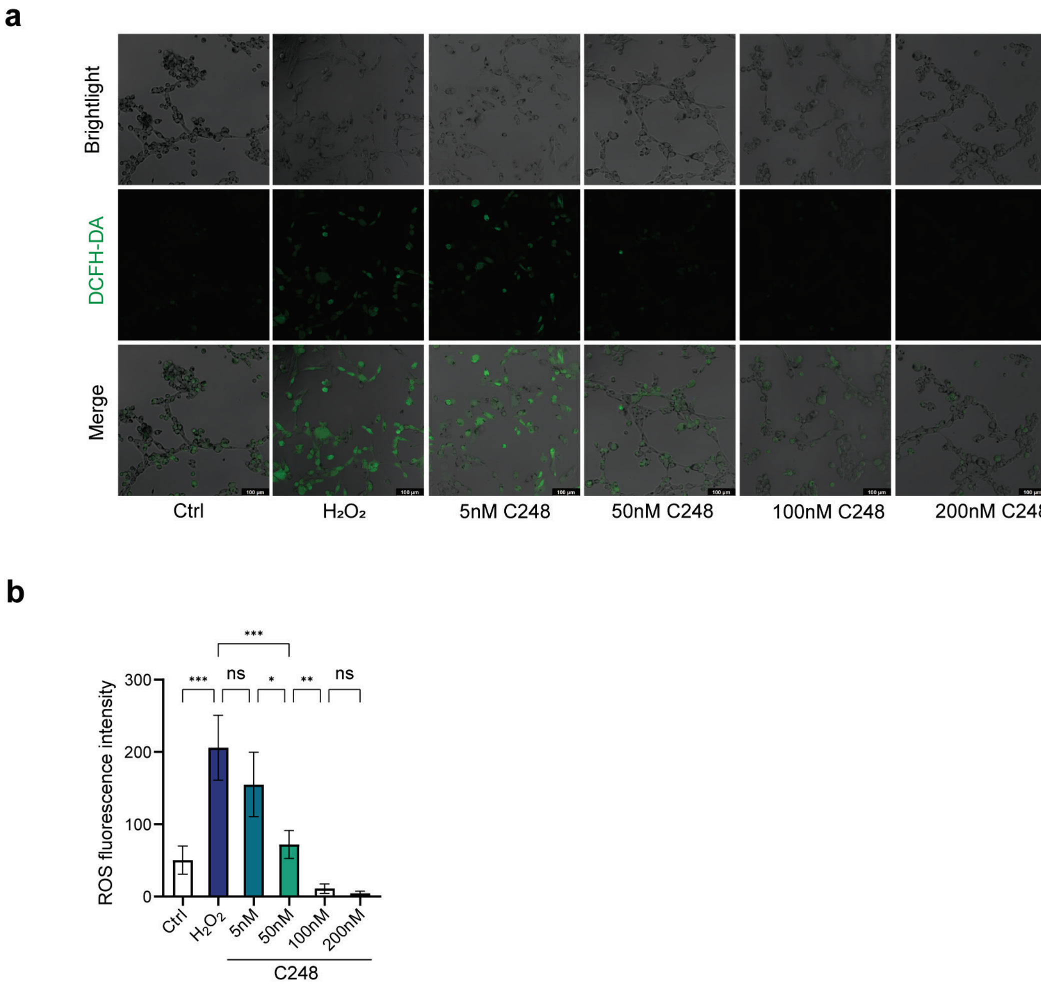

Figure S3. ROS level of LCs treated with C248. (a)Representative images of DCFH-DA staining in MLTC-1 cells treated with H_2O_2/H_2O_2+C248(5nM, 50nM, 100nM, 200nM) for 6h. All images were taken with a confocal laser scanning microscope (Leica STELLARIS5). n=3 samples per group. (b) Quantitative ROS analysis of relative DCFH-DA fluorescence intensity. n=3 samples per group.

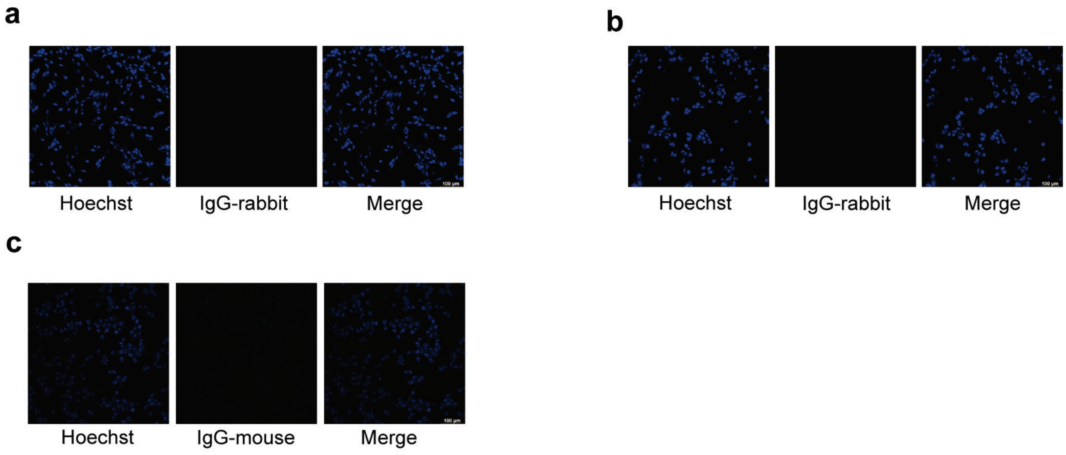

Figure S4. Negative control in immunofluorescence. (a)Representative images of IgG rabbit staining in MLTC-1 cells(negative control of P21 staining) (b)

Representative images of IgG rabbit staining in MLTC-1 cells(negative control of 3β-HSD staining). (c)Representative images of IgG muose staining in MLTC-1 cells(negative control of 4-HNE staining).

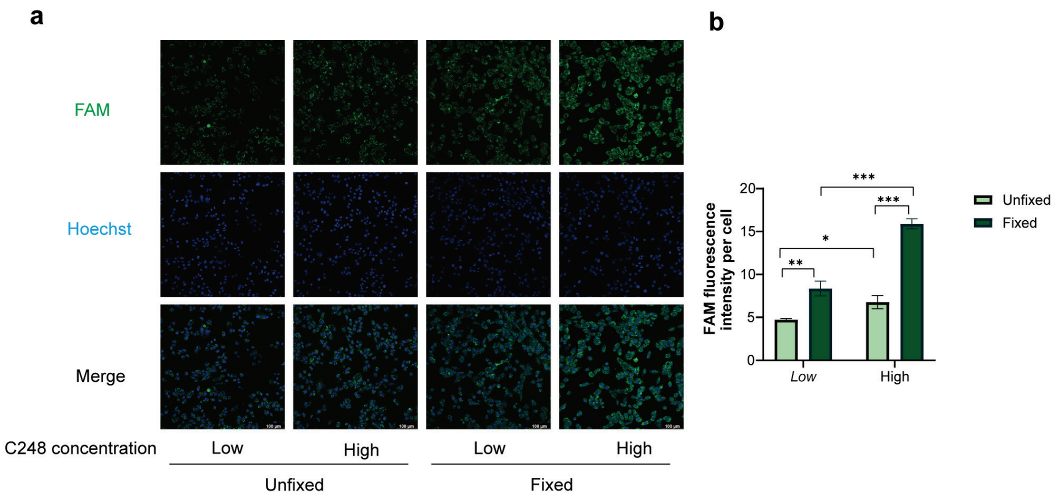

Figure S5. Negative control in immunofluorescence. (a)Representative images of FAM staining in MLTC-1 cells treated with low or high concentration about C248 and fixed or unfixed samples. (b) Quantitative analysis of relative FAM fluorescence intensity. n=3 samples per group.
